# Supplementary material for: Development and Use of a Kinetical and Real-Time Monitoring System to Analyze the Replication of Hepatitis C Virus
Source: Int J Mol Sci. 2022 Aug 5;23(15):8711. doi: 10.3390/ijms23158711 (PMC9368937; doi:10.3390/ijms23158711)
Supplement: Supplementary file 1 [file ijms-23-08711-s001.zip › Supplementary figure revised.pdf]

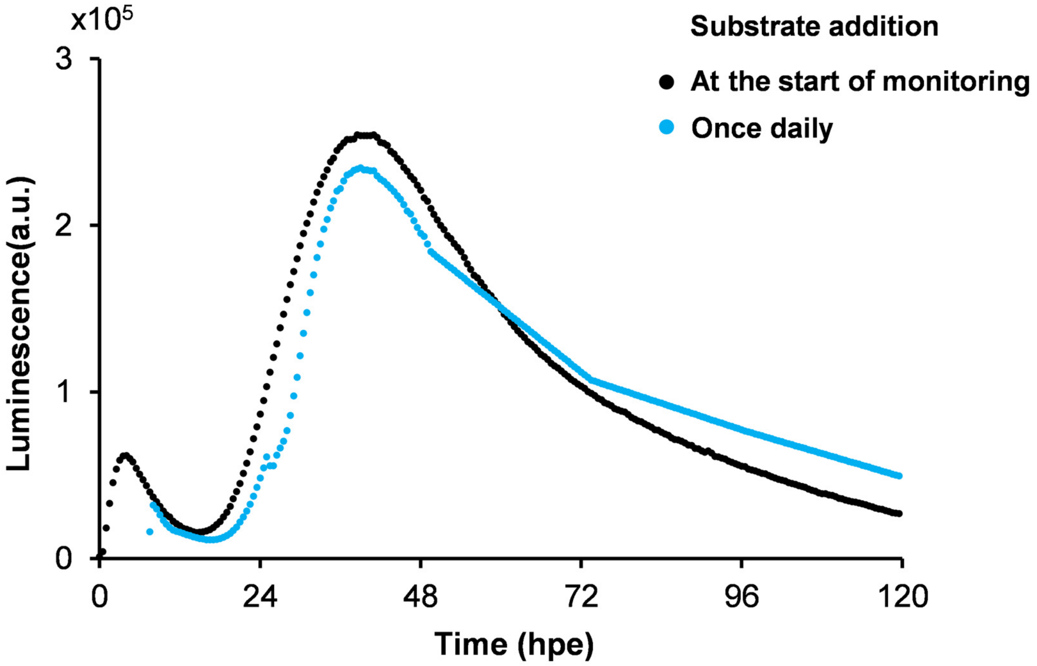

Figure S1. Comparison of luminescence signal patterns under different conditions of adding luciferase substrate**.** Huh7.5.1-8 cells were electroporated with SGR-JFH1/WT/SLG RNA. After electroporation, 3 x 10^5 cells were immediately seeded into a 35-mm dish. After 7 h, the culture medium was replaced with the medium containing 3 μM D-luciferin, followed by continuing the culture to 120 h post-electroporation (hpe) without further medium change (black line) or by continuing the culture with replacement with the D-luciferin-containing medium (as above) every 24 h (blue line).

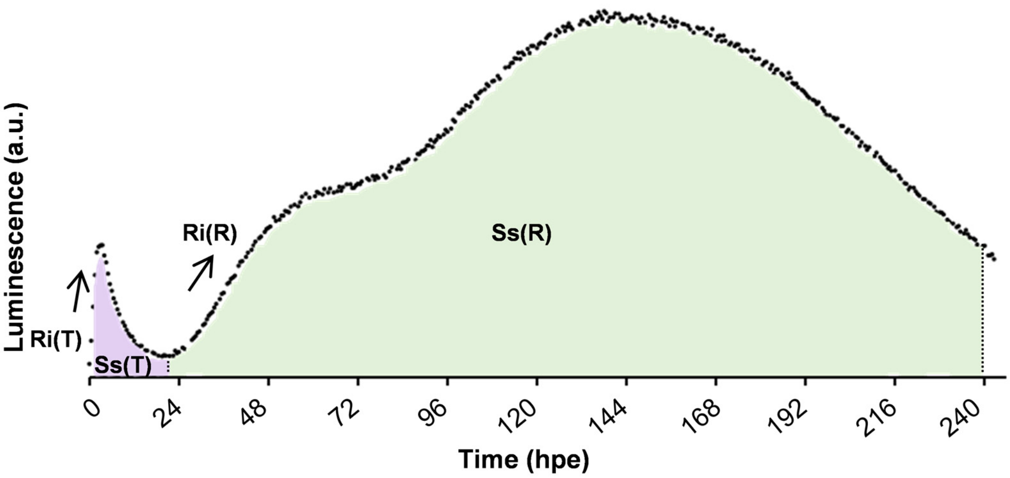

Figure S2. Indicators of translation efficiency [Ri(T) and Ss(T)] and replication efficiency [Ri(S) and Ss(R)]. HuH-7T1 cells were electroporated with SGR-JFH1/WT/SLG RNA. Luciferase activity was recorded as indicated in the legend for Figure 1B. Durations of time 0-19 and 19-216 hpe were expressed as translation phase (T) and replication phase (R), respectively. For each phase, the sum of luminescence signals (Ss(T) and Ss(R)) and the initial rates (Ri(T) and Ri(R)) were determined.

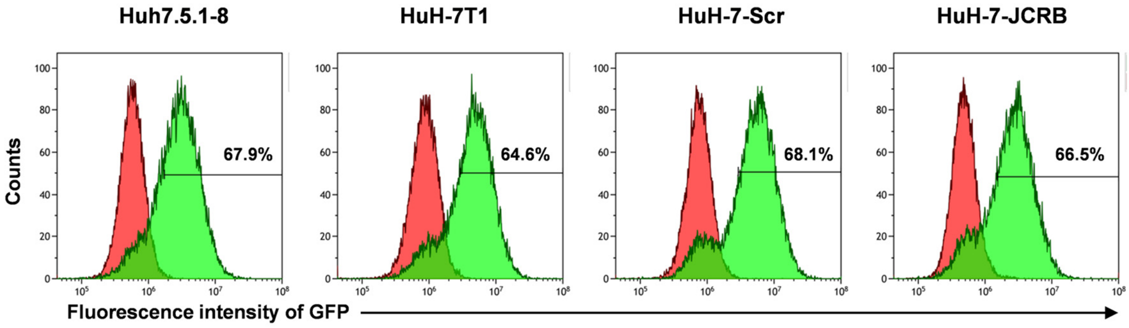

Figure S3. Determination of GFP-positive cells in cells electroporated with GFP RNA by flow cytometry**.** Huh7.5.1-8, HuH-7T1, HuH-7-Scr and HuH-7-JCRB cells were electroporated with in vitro synthesized RNA derived from pBSII-GFP. The percentage of GFP-positive cells was measured by flow cytometry after 48 h of cell culture. Green filled histograms; GFP expression, red filled histograms; isotype control.
